# Supplementary material for: Rhabdoviral Endogenous Sequences Identified in the Leishmaniasis Vector Lutzomyia longipalpis Are Widespread in Sandflies from South America
Source: Viruses. 2024 Mar 2;16(3):395. doi: 10.3390/v16030395 (PMC10974309; doi:10.3390/v16030395)
Supplement: Supplementary file 1 [file viruses-16-00395-s001.zip › viruses-2819458-supplementary.pdf]

>NCP1.1 (1413 bp)
CCCACGCCTCCTCATCAATAAAGCTCTAACGCACGAGGACGTGCGTCCTCCTAAGGAAGGAGGTGTGGCGAA
ATCGCGGAATGCTCCCAATTCCCCCAATCACCTTAACCGAAATTAGAACACAAACAATAAATTGTACCTATA
AATAAGGCAAAAATATATATTAAAATCAGTTTGGAGTTGAAAGTCAATAAGCGGAGTTTAAATTCTCTACAC
TGATACCGATATTAACAAAATGATGAGAGTTGTTGCGTATGGTTTGAGTACTGACTCACTCCAGGTACGGGA
TGCAGTTCAATTTCTATACTTATTCGGTTTGACAATCACGGAGAGGTTGGAGAAACCAGTTTATTGTTTCGGG
AAGCAGTGGTTGTCTGCCGGAACTGTTGTGTCTCCGAATATTCTCTTTAAGACAGTAAGGACAGATTACCTG
GCTGAGGAGGCAGAGCCGGTACCTCCAACTAACGTGACAGAATTGCCTGCCTTGGCTTTCCGAGTTCTTTGT
GTTTAAAGACTGCACAAGTCAAAGAATCTCGGCTATAGACTCACACTTTGGAAAACAGCAAAGTCCATGTTG
AATGCACTTACTGAGGGAGCCTCATGGAGAGGCACTGGAACCCCAGAACGATACAATACATGGGAGACATC
TAACAATTTTACAAAGTTGGTGGCCGGATATGACTGGTTCTTCCAGTTGTTTAACAAACATCAATATGCATCC
TTGAGATTCGGAACCGTTTCATCTCGAATGGCTGAGTGTACAGTCTTCAATGCATTTGAACACGTAATAGGA
ACCCTGTCGATGCCCCCTGCTCAGATTTACAAGTGGATTTGGGATCTACAGGTGATGAAGGAATACAACATT
TTTGGTAAAGAGACAGACTTCTTCGCAGAATATGGGTCTGTTTACCCATACTTCGCGTGCCTCTGTCTTTCGA
GAAAGTCCCCATATGCTATAGGCCAAGTACCTCATCTCCATACGTATTTGCATACCATTAGTTGCTTAGCAGG
GAGTCATTCATCTAAGAACGCGACTTACTACCCGGGGACCAACTTCAAAACATTAATTGGTTATGTATATTGT
ATTGTATGGATACATTGGTAACTATCCAAAAAGTGCTCTAAGTGATCGATACAAATCGATCGGAGATATTGT
GGAGGTTCTGCAAAAAAGTGTGTAGTGTCTAAATTGTGCAAGTGCCACAAAAACTCAGGCCAACTCCCCACT
GTGAGCGAATATCGCAGTGGGACTGTGAGTGAATATCACAGTGAATTATCCGGACCCACCGTGTGAATGAAT
ATCACACTTTGAAAAAGAAAAAATAGTTTGTAGAGAGCAGTGAAGTGAATCCCTAACTCTCATCTGTGAGTA
TAACCTTATCCAGTGCTTGGCCGCTCACCCAGCCATCCTGG

>NcP1.2 (950 bp)
ATGCGGCTGCaACTTCTGGAGAGTTGGAGAGCTGGCGCTGGCACATCCTCACTTAACACTTCACATTTTCACT
CACTTTCTGTCCACCGACAGCAACCAGTTCTTCCTGGTCACGTGTAGTAAATTTAAACACCACTTCTTGAGTT
CCCTCAGCAGTTACTTCAGCAACTTCTTCTTTCTTCGCATATTTGTCAATCAAGTCTTTGACAGCGGCCTCATG
TCGGACCATTCGAGCCCATCCTCCGTACGATCCAATGTAACTATCCCCTGTCATCTTGATGATGAGCTCTTTC
GCGACAAATCCGAAGCTCTTATAGTTCGTTGATCTAACAGGTCCCAAGTCTCGCTTATATCCATAATATGGGT
TATTGTTCATGGTGTTGAACGCCTGTACGAGATTCGAAAACTCTATTGGATAGAATGCCGCAATCTGATCCCA
ATCAAAATCTGAATATTCCACCATGGCTCTTCTGATAGTGCATATTGTTGTCAATCCAGAGAATGTAGtCTGG
TTCAGGATCGTCATCATCCTAATGCAGGTCGGGGGAATGTAACTCTTCAGCTTGGTGAAAAACTCCTTGGCG
GTCTCATCTGTAATGTAAATACCGAAAGTATTGTAGAAAATACGGATGACATCAACATTCAGATTCAGGGTG
ATTCCAATCTCTGATGAAAGTGCCTGTATAGTTTTGTTCAAACTGTTAGACGAAATTGAACCCCTCTTGCCAT
AAGCTAGAATCATGTTTACTGCCGCAATCGTTGCAGTAAAAGTCACATTATATCCTTTCTCTGCATACCAGTC
TCTCATTTTGATGGCATGGTCAATCGTTGTGATCTCCTGAATATCGGGATGAGCTCTCTTGAAATCTCAAGGG
TAGTTCCGTAAGTATCCAATACCTCATTGGTGTCATCGACAATTTTGTTCCGGATGGCTCAAAGCCAGAGCCG
GT

>NcP2 (1307 bp)
AAAACAGTTTTTGACAGGACAGAAGTTTATGGCTCTTAGAACTTTTTTAATACATACAAAATTTTTTCAAATG
TTTTACTTTCCGATCGCCTGATGACGTCCTTATCGTATTAGGTGCTCAGCTGGGACCGGTCCAATTATCATTGT
ATACAACGCATATCCTATAGTCCCCAATCTTAGGGGTACAAGTATGGAGACATCGTGTTTATTGCGACGCTT
AAGTTCATCATTCAATTCATATCCTCGTTTTATGTACCATATTAGTATTTCATCTGGGTCCTGTGTCCAGGGAA
CAGGTGTGGATAAATCTAGTTTGGAGGGTATAGATCCATCGCCTCCTTCATCACTCGTATTGTCAACTGACCG
AATCCTGGCCATATCCATACAACGCGACAAGTAAGTTCGTGCTTCAGAAAACCTACGCTTCAACTCATCCTG
ATTTTCATCTACCTGTACTATCATACGTCCGGCCCCAAAACCGTTTGTCCAAGCATAAACAGCGAGGGTTGCG
TAGTCTATCAATTGTTTCAAATTGATATTGGGAGGGAGGATTGCATCTTTCGAAGTGTCGCTGCAGCCTGCCA
AACACCCAATTATGTTAAACCAGGTGTGCAAAAGGGAACATTGCTCAATCCCCCATGGAGATCTCCTTGACA
TTCGTAGACATGCGAAGTATGGGAATATTGATCCTTTCTCATTTAGGAAATCACTTTCTTTCGCCATGATTAC
GTATTCATTCATCATCTGCTCATCAAATATCCATAGAGGAAGTTTATAATACGGTATAAGAGCATTGCAGAT
ATTTTTGAAGCTGTGGTAAACAGAGCACTCAGCCATCCGCGTTACGAAGCTTCCGAATCTGAACTGTGCCAA
AGGGTGATCCGGGAAAAGAACAAAAAACCAATCAAACCCAGCAACGATTTTGGCAAAGTTATCATTGTTCG
GCCAGCCATTGTAGGAGTTTGGGGTTGCTGTACCTCTCCATTCTCCGTTGATCTCTAGCTGTCTCATATACGTT
TGAGCCCTACGTTGCAGAGAGGCTTTGTGGGTGTCATTTTTTGTCTTATGTACTCGATAGATGCATATAATCC
TGAAGGCGAGTGAAGCATTTTCGTTCACAAGAAGTGGTGGATTGGGATCCACATTATCAAATACATAATCAT
TGAACTCAATATTGAACGGCGTAAGGGGGGTCACTTTCTCACCCTTTTTAAGCCATGGTTTGCCAAAACAATT
AATGTCCTTCGGACATTCCTTTTCCATTTCGAGACCAAACATGTAGAGAAAGTGGACAGCTTCCTGGATTT

Figure S-1: Extended nucleotide sequences of previously identified EVEs NcP1.1 and NcP2. The original sequences, initially identified in the exosomal fraction, are written and underlined in red. The EVE NcP1.2 identified *in silico* is also represented.
